# Dietary Patterns and Risk of Age-Related Cognitive Decline and Dementia: Enthusiasm Before Evidence?

**DOI:** 10.1093/geroni/igaf122.1872

**Published:** 2025-12-31

**Authors:** Sarah Booth

**Affiliations:** Tufts University, Medford, Massachusetts, United States

## Abstract

With respect to brain aging, there has been particular emphasis in the literature on three named diet patterns, DASH, the “Mediterranean” diet, and the MIND diet. Between 1980 and 2024, 139 human observational or intervention studies were published that examined the role of these individual dietary patterns in brain aging. When systematically reviewed using the USDA Nutrition Evidence Systematic Review method and an a priori established protocol, a common theme emerged: diets characterized by higher consumption of vegetables, fruits, legumes, nuts, fish, seafood, and unsaturated vegetable oils, alongside lower intake of red and processed meats and sugar-sweetened beverages, are consistently associated with a reduced risk of cognitive decline and dementia. However, a significant knowledge gap remains regarding the critical timing and duration of dietary pattern consumption in early adulthood for the prevention of cognitive decline. Furthermore, the biological mechanisms responsible for the cognitive protective effects of these diets remain unclear. Ultimately, it is important to understand the underlying mechanisms of select nutritional factors found within healthy dietary patterns that are not sufficiently consumed in the U.S. population yet show promise in delaying various aspects of brain aging.

